# Efficient Generation of Human Embryonic Stem Cell-Derived Corneal Endothelial Cells by Directed Differentiation

**DOI:** 10.1371/journal.pone.0145266

**Published:** 2015-12-21

**Authors:** Kathryn L. McCabe, Noelia J. Kunzevitzky, Brian P. Chiswell, Xin Xia, Jeffrey L. Goldberg, Robert Lanza

**Affiliations:** 1 Ocata Therapeutics, Marlborough, MA, 01752, United States of America; 2 Bascom Palmer Eye Institute, University of Miami School of Medicine, Miami, FL, 33136, United States of America; 3 Emmecell, Key Biscayne, FL, 33149, United States of America; 4 Shiley Eye Institute, University of California San Diego, La Jolla, CA, 92093, United States of America; 5 Byers Eye Institute, Stanford University School of Medicine, Palo Alto, CA, 94303, United States of America; Instituto Butantan, BRAZIL

## Abstract

**Aim:**

To generate human embryonic stem cell derived corneal endothelial cells (hESC-CECs) for transplantation in patients with corneal endothelial dystrophies.

**Materials and Methods:**

Feeder-free hESC-CECs were generated by a directed differentiation protocol. hESC-CECs were characterized by morphology, expression of corneal endothelial markers, and microarray analysis of gene expression.

**Results:**

hESC-CECs were nearly identical morphologically to primary human corneal endothelial cells, expressed Zona Occludens 1 (ZO-1) and Na^+^/K^+^ATPase**α**1 (ATPA1) on the apical surface in monolayer culture, and produced the key proteins of Descemet’s membrane, Collagen VIII**α**1 and VIII**α**2 (COL8A1 and 8A2). Quantitative PCR analysis revealed expression of all corneal endothelial pump transcripts. hESC-CECs were 96% similar to primary human adult CECs by microarray analysis.

**Conclusion:**

hESC-CECs are morphologically similar, express corneal endothelial cell markers and express a nearly identical complement of genes compared to human adult corneal endothelial cells. hESC-CECs may be a suitable alternative to donor-derived corneal endothelium.

## Introduction

Disease and injury to the cornea are leading causes of blindness worldwide. The gold standard treatment for many corneal diseases relies on surgical replacement with cadaveric corneas. In countries with well-established eye banks to collect and distribute healthy donated corneal tissue, corneal transplantation may be routinely performed, but in countries without such a system, millions of people are left visually impaired or blind due to lack of available donor corneas [1]. Even with improved eye banking, there is limited availability of high quality donor corneas [2]. Therefore it is critical to pursue alternative approaches that do not rely on donor corneas.

The cornea consists of three cellular layers which are necessary for vision. Defects in any of these layers will result in absence of or reduced visual acuity. The innermost layer, the corneal endothelium, is comprised of a monolayer of corneal endothelial cells (CECs) that keeps the cornea relatively dehydrated so the stroma does not become opaque [3]. Thus well-functioning corneal endothelium is critical for the overall health of the cornea and visual acuity of the patient. Corneal endothelium quality decreases naturally with age, as dead cells are not replaced, and remaining cells expand in size to maintain the monolayer, but functionality is eventually impaired [4]. Surgeries including cataract extraction and corneal transplantation itself also result in significant CEC loss, thus motivating clinicians to select donor corneas with the highest possible initial density of CECs when transplant is required. A recent study has calculated an increasing cost of donor corneas as surgeons’ preference for younger corneas with higher CEC density becomes more difficult to supply [2].

Recent advances in surgical techniques for corneal transplantation which transplant only the corneal endothelium and some stroma (DSEK) and modifications of this technique (DMEK), have lent support to the premise of transplanting a tissue culture-engineered corneal endothelium [5]. Recent progress has been made in culturing primary adult human corneal endothelial cells (HCECs) [6]; however, it remains attractive to mass produce CECs for transplantation. Therefore, we sought to derive corneal endothelium from human embryonic stem cells (hESCs) to produce hESC-derived corneal endothelial cells (hESC-CECs) in large, reproducible batches.

## Materials and Methods

### hESC-CEC and Primary HCEC Culture

hESC lines H1 Oct4 eGFP (WiCell, [7]), H9 (WiCell, [8]), Ma09 [9] and NED07 [10],were cultured feeder-free on hESC-qualified matrigel- (BD Biosciences) coated 6 well plates (Falcon) with mTESR1 media as directed by the manufacturer (Stem Cell Technologies) with the exception of using Cell Dissociation Buffer (Thermo Fisher Scientific) for 5–6 minutes at 37°C for the passaging of cells approximately 1:10 every 4–5 days. The induction of neural crest began on the day before or the day of normal passaging of hESC. Control hESC mRNA were collected at this time. We have adapted a previously published protocol [11] to generate corneal endothelial cells. hESC were exposed to the dual Smad inhibitors, 500 ng/ml Noggin and 10 mM SB431542, starting on Day 0 for 3 days (Day 0-Day 2) in a basal media of 80% DMEM-F12 (Thermo Fisher Scientific), 20% knock out serum replacement (Thermo Fisher Scientific), 1% non-essential amino acids (Thermo Fisher Scientific), 1 mM L-glutamine (Thermo Fisher Scientific), 0.1mM b-mercaptoethanol (Sigma), and 8 ng/ml FGF2 (Peprotech) (together, “dual Smad induction media”). On day 2, Dual Smad induction media was replaced with “cornea media”, containing the same basal components with the addition of 0.1X B27 supplement (Thermo Fisher Scientific), 10 ng/ml human recombinant PDGF-BB (Peprotech), and 10 ng/ml recombinant mouse Dkk-2 (R&D Systems). On Day 3, presumptive hESC-CECs could either be maintained in cornea media daily (original method) for 7 days, or transferred to a new matrigel-coated well. To transfer the presumptive corneal endothelial cells, we used Cell Dissociation Buffer (Thermo Fisher Scientific) for 8–9 minutes at 37°C and resuspended in cornea media. Presumptive hESC-CECs were fed daily for at least 7 additional days and then collected for analyses. Phase contrast photographs were taken using a Nikon TE2000-S microscope, Spot 7.2 Color Mosaic Camera with Spot Advanced 4.6 software. Minor alterations of the images were processed using Adobe Photoshop CS4. The methodology for generating hESC-CECs can also be found in the patent application W02013086236A3.

Human adult cadaveric donor corneas were obtained from the Lions Eye Institute for Transplant and Research (Tampa, FL) and they were handled in accordance with the Declaration of Helsinki. Informed written consent was obtained by the eye bank for the use of tissue for research. This work was conducted with the approval of the University of Miami Ethics Committee.

Human adult primary corneal endothelial cells (HCECs) were isolated and cultured following previously published protocols [12,13]. Briefly, Descemet’s membrane and the endothelium were peeled off donor corneas, incubated overnight in basal media and 8% FBS (Hyclone) and dissociated into a cell suspension the next day after a 1 hour incubation at 37°C in 5% CO_2_ with 0.02% EDTA (Sigma Aldrich, St Louis, MO). HCECs were then seeded in a culture plate that had been pre-coated with FNC (US Biologicals) in growth medium containing 8% FBS, 5ng/ml epidermal growth factor (EGF), 20ng/ml nerve growth factor (NGF) (PeproTech, Rocky Hill, NJ), and 100μg/ml of bovine pituitary extract (Biomedical Technologies, Stoughton, MA).

For immunostaining, cells were transferred on Day 3 onto 12 mm #1 glass coverslips (Chemglass Life) and cultured as described above. Glass coverslips were coated with poly-d-lysine hydrobromide (PDL) MW 30–70K dissolved in water to a final concentration of 50 mg/mL at 37°C for 15–30 minutes, rinsed at least 3 times for 5 minutes each, and air-dried. After PDL coating, coverslips were coated in Matrigel (BD Biosciences) as previously described [14].

### Immunostaining and Cell Counting Statistics

For immunostaining of the expression of Zona Occludens protein 1 (ZO-1), von Willebrand factor (vWF), p75/NGFR and CD31, traditional methods were utilized. Cells were fixed onto glass coverslips for 15 minutes at room temperature with 4% paraformaldehyde (Boston Bioproducts), rinsed with phosphate buffered saline (PBS) (Boston Bioproducts), and permeabilized on ice for 5 minutes with 0.2% Triton X-100 (Sigma) and 1% goat serum (Jackson Laboratories) in PBS. Coverslips were subsequently blocked for at least 1 hour at room temperature in 10% goat serum and 1% bovine serum albumin (BSA) (Jackson Laboratories) in PBS. Mouse anti-ZO-1 (1:100, Thermo Fisher Scientific), mouse anti-CD31 (1:100, Dako), rabbit anti-vWF (1:1000; Dako), Mouse anti-p75/NGFR (1:100 Advanced Targeting Systems) or directly conjugated anti-ZO-1-Alexa 596 or -FITC (1:100; Thermo Fisher Scientific) were diluted with 1% BSA in PBS and incubated in a humidified chamber at 4°C overnight. If using directly conjugated primary antibody, coverslips were then rinsed in PBS at least 3 times for 15 minutes each and then exposed to DAPI to visualize nuclei. If unconjugated, coverslips were rinsed in PBS at least 3 times, 15 minutes each. Secondary antibody donkey anti-mouse Alexa 555 (Thermo Fisher Scientific) was diluted 1:1000 in 1% BSA in PBS and incubated at room temperature for 1.5 hours. Coverslips were rinsed in PBS at least 3 times 15 minutes each and then exposed to 2 mg/ml DAPI (Sigma) in PBS for 5–10 minutes at room temperature to counterstain nuclei. Coverslips were then rinsed in PBS for at least 3 times for 15 minutes each, with a final quick rinse in distilled water before mounting on a slide with Permafluor (Thermo Scientific). Fluorescently labeled cells were imaged using an Olympus Bx51 microscope, Qimaging QICam camera with QCaputure Pro 6.0. Minor alterations of the images were processed using Adobe Photoshop CS4. For determining the density of hESC-CECs, the cells were stained with ZO-1 to confirm identity and density. From two separate experiments, three random 40X fields were counted (mean ± SEM).

For immunostaining of Na^+^/K^+^ATPase**α**1, a slightly different method was used as described [15]. Coverslips were washed with PBSCM (PBS + 1mM CaCl2 + 1mM MgCl2). Cells were permeabilized with cold 100% MeOH at -20°C for 20 minutes. Coverslips were then rinsed 3 times, 5 minutes each with PBSCM. Coverslips were next blocked for at least 1 hour at room temperature with fluorescent detection buffer (FDB) containing 5% FBS (Atlas), 5% donkey serum (Jackson Laboratories), and 2% BSA in PBSCM. Mouse anti- Na^+^/K^+^ATPase**α**1 clone C464.4 (Millipore) was diluted 1:100 in FDB and incubated in a humidified chamber overnight at 4°C. Coverslips were washed at least 3 times for 15 minutes each at room temperature with PBSCM before incubating in secondary donkey anti-mouse IgG antibody conjugated with rhodamine red (Jackson laboratories), diluted 1:100 in FDB for 1.5 hours at room temperature. Coverslips were washed at least 3 times for 15 minutes each at room temperature with PBSCM before adding DAPI and mounting and imaging as previously described.

### Western Blot

hESC-CECs were grown as described above for 7 days then lysed from the subcellular extracellular matrix (ECM) using ammonium hydroxide as described previously [16]. This allowed removal of the CECs without damaging the ECM that was secreted below the cells. After exposure to ammonium hydroxide, the plate was washed three times with PBS to dislodge any remaining cells. Reducing sample buffer was then added to collect the ECM protein. Standard Western blot analysis and image analysis (Biorad) was used to assess for ECM proteins COL8A1 and COL8A2. Antibodies to COL8A1 and COL8A2 were a kind gift from Nicholas Greenhill, Wellington School of Medicine, Wellington, New Zealand [17].

### RNA Isolation, cDNA Synthesis, and Quantitative PCR (QPCR) and Statistics

Before collecting RNA, cells were rinsed with Dulbecco’s PBS (Thermo Fisher Scientific). RNA was collected and purified using RNAqueous or RNeasy RNA extraction kits (Thermo Fisher Scientific and Qiagen). After isolation, potential genomic DNA was removed by Turbo DNAse (Thermo Fisher Scientific) or DNAse (Qiagen) as per manufacturer’s directions. cDNA was synthesized from 1 mg of total RNA using the SuperScript^®^ III First-Strand Synthesis SuperMix for qRT-PCR (Thermo Fisher Scientific). cDNA was diluted 1:30 in water to be in the middle of the five-fold dilution range for 100% efficiency.

QPCR was performed using Taqman Fast Advanced Master Mix (Thermo Fisher Scientific) on the Step One (Applied Biosystems) or SSO Fast Master mix (Biorad) on the CFX96 Connect Real Time System (Biorad) with the following cycling parameters: Initial 95°C for 30 seconds, then 40 cycles of 95°C for 5 seconds and 60°C for 10 seconds. Taqman Probes (Thermo Fisher Scientific) were as follows in Table 1. QPCR data analysis and calculation of error bars was completed using the software and standard settings provided by the manufacturer of the Step One Software (Applied Biosystems) and CFX Manager Software (Biorad). Relative mRNA levels were determined by comparing naïve hESCs using the **ΔΔ**Ct method [18].

**Table 1:** QPCR probes used in study.

Gene Name	Gene Symbol	TaqMan^®^ Gene expression Assay Reference
18S ribosomal RNA	*18S rRNA*	HS99999901_s1
adenylate cyclase 10	*ADCY10*	Hs01037975_m1
aquaporin 1	*AQP1*	Hs00166067_m1
ATPase, Na+/K+ transporting, alpha 1 polypeptide	*ATP1A1*	Hs00167556_m1
ATPase, Na+/K+ transporting, alpha 3 polypeptide	*ATP1A3*	Hs00265163_m1
carbonic anhydrase XII	*CA12*	Hs01080902_m1
carbonic anhydrase II	*CA2*	Hs00163869_m1
carbonic anhydrase IV	*CA4*	Hs00426343_m1
cystic fibrosis transmembrane conductance regulator	*CFTR*	Hs00357011_m1
collagen, type VIII, alpha 1	*COL8A1*	Hs00156669_m1
collagen, type VIII, alpha 2	*COL8A2*	Hs00697025_m1
cysteine/tyrosine-rich 1	*CYYR1*	Hs00364793_m1
forkhead box C1	*FOXC1*	Hs00559473_s1
glypican 4	*GPC4*	Hs00155059_m1
keratin3	*Krt3*	Hs00559942_m1
keratocan	*Kera*	Hs00559942_m1
lumican	*Lum*	Hs00158940_m1
Nanog homeobox	*NANOG*	Hs02387400_g1
nerve growth factor receptor*NGFR*Hs00609976_m1paired-like homeodomain 2	*PITX2*	Hs00165626_m1
POU class 5 homeobox 1 (OCT4)	*POU5F1 (OCT4)*	Hs03005111_g1
solute carrier family 16 (monocarboxylate transporter), member 1	*SLC16A1*	Hs00161826_m1
solute carrier family 16 (monocarboxylate transporter), member 3	*SLC16A3*	Hs00358829_m1
solute carrier family 16 (monocarboxylate transporter), member 7	*SLC16A7*	Hs00940850_m1
solute carrier family 4 (anion exchanger), member 2	*SLC4A2*	Hs01586776_m1
solute carrier family 4 (sodium bicarbonate cotransporter), member 4	*SLC4A4*	Hs00186798_m1
solute carrier family 4, sodium borate transporter, member 11	*SLC4A11*	Hs00984689_g1
solute carrier family 9, subfamily A (NHE1, cation proton antiporter 1), member 1	*SLC9A1*	Hs00300047_m1
SRY (sex determining region Y)-box 10	*SOX10*	Hs00366918_m1
von Willebrand factor	*VWF*	Hs00169795_m1

### Microarray and Gene Expression Analysis and Statistics

hESC-CECs were grown in three separate biological replicate experiments as previously described but with an extra week of growth. Adult HCECs were isolated from three cadaveric donor corneas and expanded in culture as previously described [12,13]. HCECs were harvested at confluency, flash frozen in liquid N_2_ and RNA was isolated using RNeasy RNA isolation kit (Qiagen) with an additional DNAase treatment step (Qiagen) following the manufacturer’s standard protocols. RNA samples were further processed for hybridization to Affymetrix’ Human Exome ST arrays at the University of Miami Genomics Core Facility. Raw data were first normalized and analyzed using Genespring GX12 software (Agilent Technologies, Santa Clara, CA). After normalization, probes were filtered using 2 different criteria: expression values between 20 and 99^th^ percentile across all probes and at least 2 out of the 3 samples in at least one condition passed these criteria. 28374 out of 28869 total probes (98%) were used for subsequent analysis with Excel (Microsoft Corporation, Redmond, WA). Gene expression levels were compared using an unpaired t-test with asymptotic p-value comparison. Multiple testing correction was done with Benjamini-Hochberg false discovery rate method.

For comparison of gene expression between hESC-CECs and other cell types, we used raw microarray file datasets for the same platform downloaded from the NCBI’s Gene Expression Omnibus (GEO) repository that included: human umbilical vein endothelial cells in culture from passage 1 or 2 (series GSE23909, 5 samples from 5 individuals [19]), uncultured human pancreatic islet cells (samples GSM762810, -11 and -12, from 3 individuals [20]), and HEK-293 cells (series GSE36575, 3 control samples [21]). HCEC and hESC-CEC microarray raw data files have been loaded into GEO accession number GSE70954.

## Results

### Two-Step Generation of hESC-CECs

**Fig 1:**
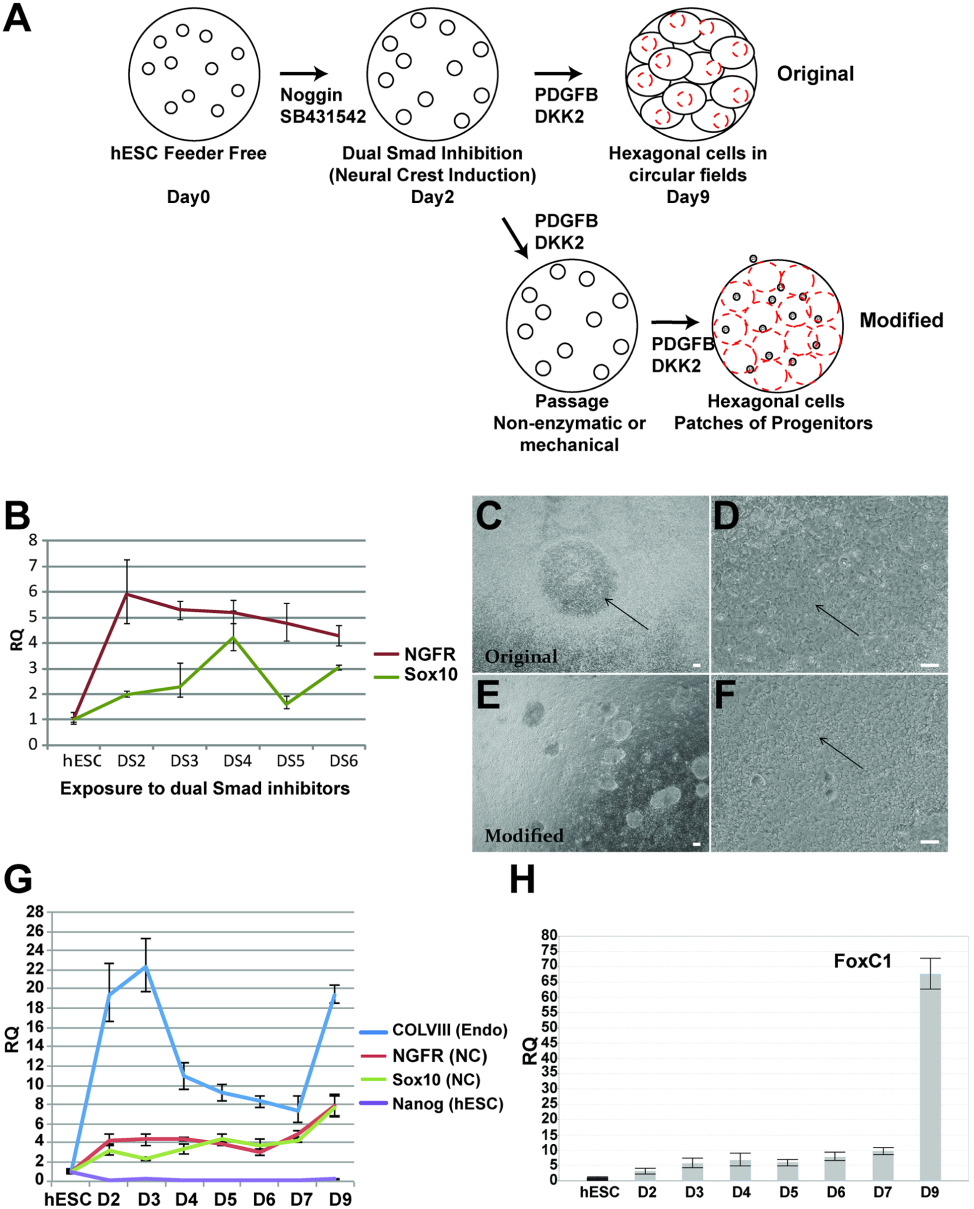
Generation of corneal endothelial cells (CEC) from human embryonic stem cells (hESC). A. Diagram outlining the process of inducing feeder free hESC with Noggin and SB431542 (dual Smad inhibitors) to become neural crest. Neural crest cells were then exposed to PDGFB and DKK2 to induce neural crest to differentiate into neural crest. Passaging cells on Day 3 improved the process. By Day 10, hexagonal/polygonal corneal endothelial cells were present in large numbers, with some progenitors remaining as clumps. B. Neural crest markers NGFR and Sox10 mRNA were expressed after 2 days of exposure to dual Smad inhibitors as determined by QPCR normalized to hESC mRNA levels of endogenous control, PGK1. C. Using 2 day method, low power phase contrast picture of corneal endothelial cells one week after induction. Center CEC colony was surrounded by progenitor-like cells. D. Using 2 day method, higher power phase contrast picture of corneal endothelial cells one week after induction. E. Using day3 dissociation method, low power phase contrast picture of corneal endothelial cells one week after induction. Notice that the CEC colony is a continuous sheet with some progenitor-like colonies. F. Using day3 dissociation method, higher power phase contrast picture of corneal endothelial cells one week after induction. G. COL8A1 mRNA, a marker for CEC, was expressed as early as 2 days of differentiation. NGFR and Sox10 mRNA, markers of neural crest, continue to be expressed during differentiation. Nanog mRNA, a hESC maker, is not detectable after 2 days of differentiation. See Fig 2A for more detail. H. FoxC1, a marker of neural crest and potentially corneal endothelium, can be detected after 2 days of differentiation, with peak levels at Day9, similar to NGFR and Sox10. QPCR normalized to hESC mRNA levels of endogenous control, PGK1. Scale bars = 10 **μ**m. RQ = relative quantification, Endo = corneal endothelium, NC = neural crest.

During development, the corneal endothelium arises from cells that originate from neural crest. The neural crest is a multipotent population of progenitors that gives rise to a large number of the diverse differentiated cell types including neurons, glia, connective tissue, bone, the corneal endothelium and stroma, and more [22]. To generate hESC-CEC cultures in vitro, we strove to recapitulate the development of neural crest into CECs. The first step was to convert hESCs into multipotent neural crest in large quantities, and the second to convert the neural crest cells into CECs (Fig 1A). There were several robust methods for generating neural crest from hESC, many which either require feeder cells or an embryoid body stage. However, we modeled our neural crest derivation after a rapid, simple protocol adaptable to feeder-free hESCs [11]. This protocol generates neural crest and central nervous system (CNS) neural progenitors by exposing the hESC to the dual Smad inhibitors SB431542 and Noggin for up to 11 days. Interestingly, longer exposures to SB431542 and Noggin resulted in more CNS progenitors than peripheral nervous system (PNS)-derived neural crest. The authors show the presence of p75 could be detected as early as day 3, which led us to speculate if a shorter incubation period with the dual Smad inhibitors would be more cost and time effective while still generating neural crest. To test this hypothesis, hESCs were exposed to the dual Smad inhibitors starting on Day 0 and RNA was collected from Day 2 to Day 6. The expression of two neural crest markers, NGF receptor (NGFR/p75/CD271) and Sox10 were analyzed by QPCR. Relative levels were determined by comparing naïve hESCs using the **ΔΔ**Ct method [18]. As early as Day 2, Sox10 was upregulated 2 fold and NGFR 6 fold over hESC, indicating the differentiation into neural crest had already begun (Fig 1B). This was 6 days earlier than previously reported [11]. Because neural crest genes were upregulated by day 2, and because such short exposure time would be unlikely to generate CNS progenitors based on previously published data [11], this time point was chosen for subsequent experiments.

**Fig 2:**
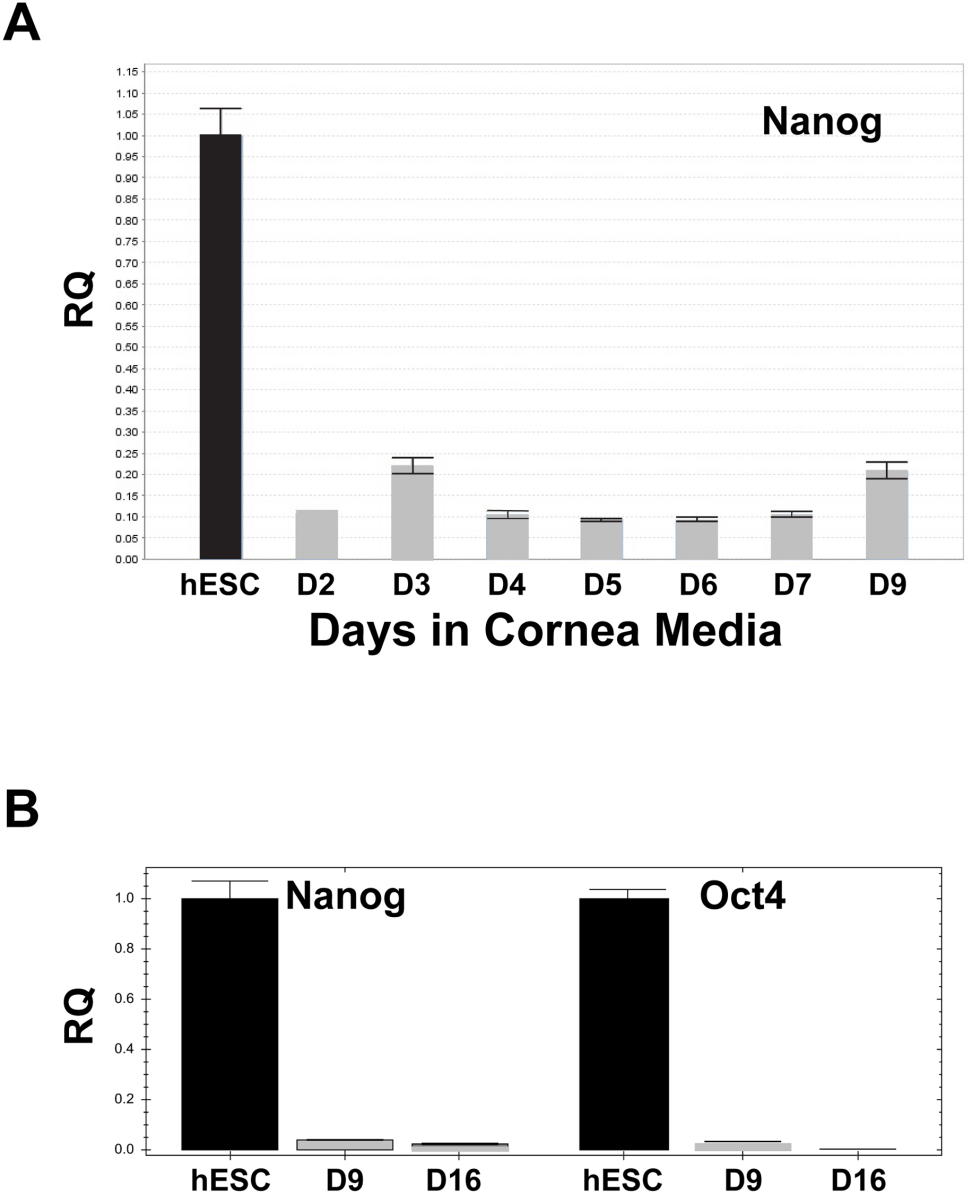
hESC markers was greatly reduced after 2 days of neural crest differentiation. A. Nanog, a marker for pluripotency, was expressed at 0.1–0.2x compared to hESC from Day 2–9. B. Nanog and another pluripotency marker, Oct4, levels were expressed at extremely low levels at Day 9 and Day 16 of cornea culture.

The second step was to induce the neural crest to become corneal endothelium. In order to generate a list of candidate inducers, we analyzed the published expression patterns of factors expressed by the lens, corneal epithelium, and the periocular neural crest. Some of the factors tested included: angiopoietin like protein 7 (ANGLPT7), epidermal growth factor (EGF), fibroblast growth factor 2 (FGF2), transforming growth factor beta 2 (TGF**β**2), platelet derived growth factor b (PDGFB), and dickkopf related protein 2 (DKK2). Of these, we found that PDGFB and DKK2 (in the presence of FGF2) were able to generate flat circular colonies of hexagonal/polygonal cells within the confluent progenitors surrounding in a raised circle, making them easy to identify morphologically. The hexagonal/polygonal colonies could be detected within 5 days of exposure to PDGFB and DKK2, and were readily seen after 1 week (Fig 1C–1F); negative data not shown for ANGLPT7, EGF, and TGF**β**2). The progression of the cells from hESC to neural crest to CECs was analyzed by QPCR markers for hESCs (Nanog, Oct4), neural crest (NGFR, Sox10, FoxC1), and CECs (COL8A1), with PGK1 as the endogenous control. hESCs were exposed to the dual Smad inhibitors from day 0 and changed to the cornea media at day 2. Cells were collected almost daily through day 9. As expected, the hESC marker Nanog was expressed at extremely low levels after day 2 of exposure to dual Smad inhibitors (Fig 1G). The low expression of Nanog from Day 2- Day9 can be seen in greater detail in Fig 2A. Not surprisingly, both Nanog and another key hESC marker, Oct 4 are expressed at very low levels at Day 9 and Day 16 of cornea culture (Fig 2B). As seen before, neural crest markers were present after day 2 of dual Smad inhibitors. However, when analyzing the progression of neural crest makers the levels remained relatively steady until an increase at day 9, suggesting that neural crest progenitors were maintained in the culture and might be proliferating after a week in culture. Alternatively, the newly derived hESC-CEC could also be expressing NGFR and Sox10, as there was evidence adult primary CECs express p75/NGFR [23]. An additional marker for neural crest [24] thought to be also expressed by corneal endothelium [25], FoxC1 is 3–5 fold upregulated from days 2–7 of cornea culture, but becomes highly expressed at day 9 (Fig 1H). Collagen8A1 was expressed by hESC-CECs and could be detected as early as day 2, indicating that hESC-CECs were beginning their differentiation. Relative levels of Collagen8A1 decreased for days 4–7, but were highly expressed by day 9 (1 week after induction). Taken together, these data suggest that hESC-CECs could be generated by inducing hESCs to become neural crest, and subsequent exposure to growth factors PDGFB and DKK2 in the presence of FGF2 drove the cells to become hexagonal/polygonal CEC-like progeny that express COL8A1 mRNA.

**Fig 3:**
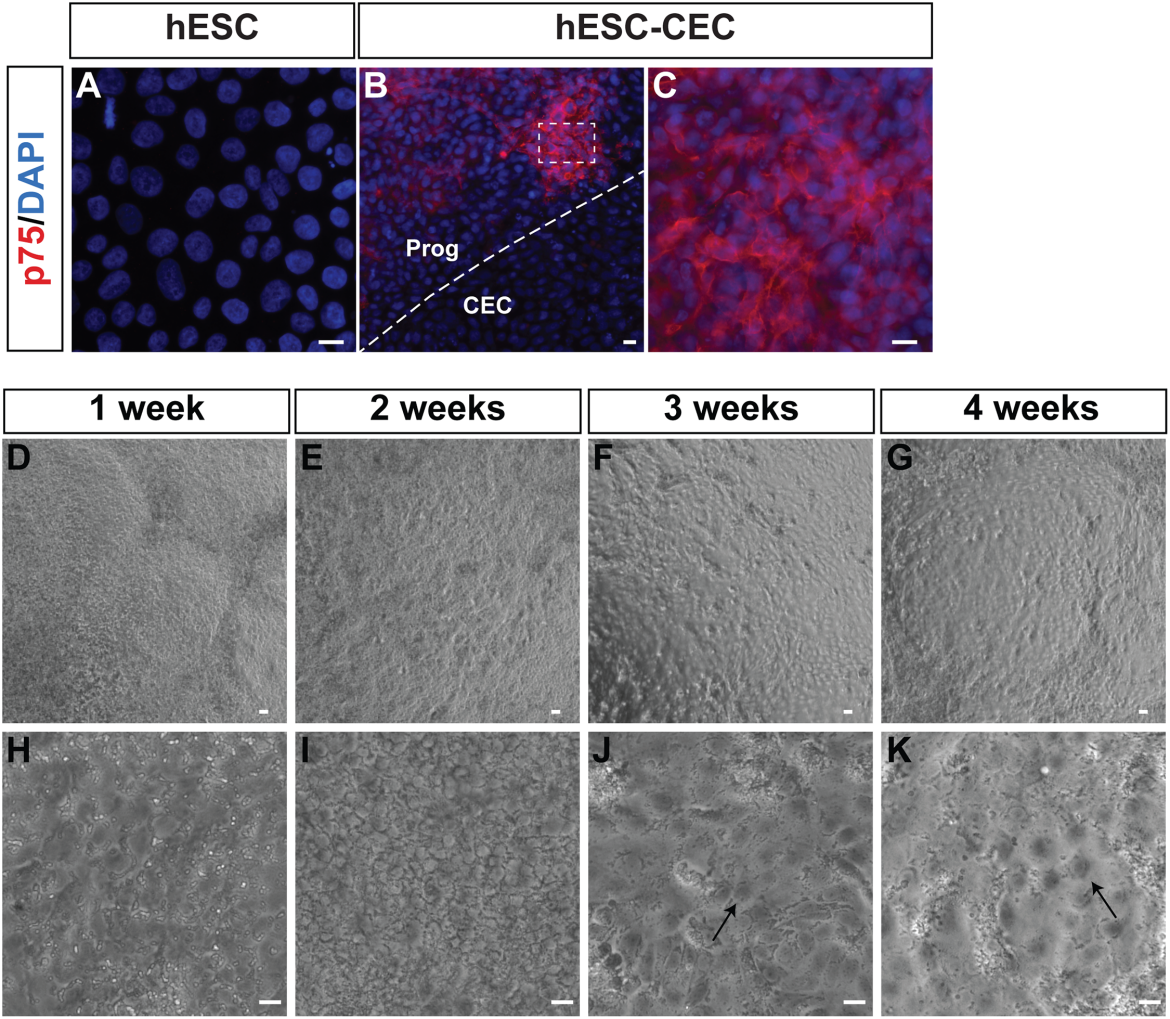
Improvement in morphology over 4 weeks of culture with 3 day transfer method. A. hESC did not express p75 (red). Nuclei were visualized using DAPI (blue). B. Using the 2 day culture method, raised progenitor-like colonies expressed the neural crest marker, p75 (red). Nuclei were visualized using DAPI (blue). Dotted line indicates border between hESC-CEC and progenitor-like colony. C. Higher power magnification of boxed area in B shows expression of p75 in progenitor-like cells adjacent to the hESC-CEC. D-K. hESC-CEC were generated using 3 day transfer method. Phase contrast pictures were taken from week 1–4 of culture. By week 3–4, the hexagonal hESC-CEC are tightly packed and more uniform in appearance. Scale bar = 10 **μ**m.

In our initial studies, morphological examination and QCPR analysis suggested that the cells in culture were not pure CECs. Many of the progenitor-like colonies were found to express the neural crest marker, p75 (Fig 3A–3C). The dotted line outlines the border between the hexagonal hESC-CEC and the progenitor-like cells. Note that hESC are not expressing p75 (red, Fig 3A). Therefore, we set out to improve the method by removing contaminant cells by nonenzymatic digestion. The hypothesis was that differentiated contaminant cells would likely be tightly adhered to the plate and therefore not be transferred. To test this hypothesis, neural crest cells were transferred to new wells on various days after the hESC were exposed to dual Smad inhibitors from day 0–2. Induced neural crest days were dissociated on day2, 3, 4, 5, or 6 and then transferred to a new well. Although corneal endothelial cells were generated from all experiments (data not shown), the optimal time was dissociation on day3 as determined by morphology and expression of Col8A1 mRNA. This resulted in the apparent ratios (determined visually) of corneal endothelial cells and progenitor cells being switched, such that in the improved method, the corneal endothelial cells formed a sheet of cells with some three dimensional progenitor-like colonies (Figs 1E, 1F and 3D–3K). The detail of the morphological changes over time can be observed by week 3 and 4, the hESC-CEC were tightly packed with a cobblestone appearance with few progenitor-like colonies (Fig 3H–3K). Interestingly, these progenitor-like colonies could be manually dissected from the culture (data not shown) or would disappear with extended culture (Fig 3G and 3K).

### Expression and Apical Localization of Corneal Endothelial Proteins by hESC-CECs

**Fig 4:**
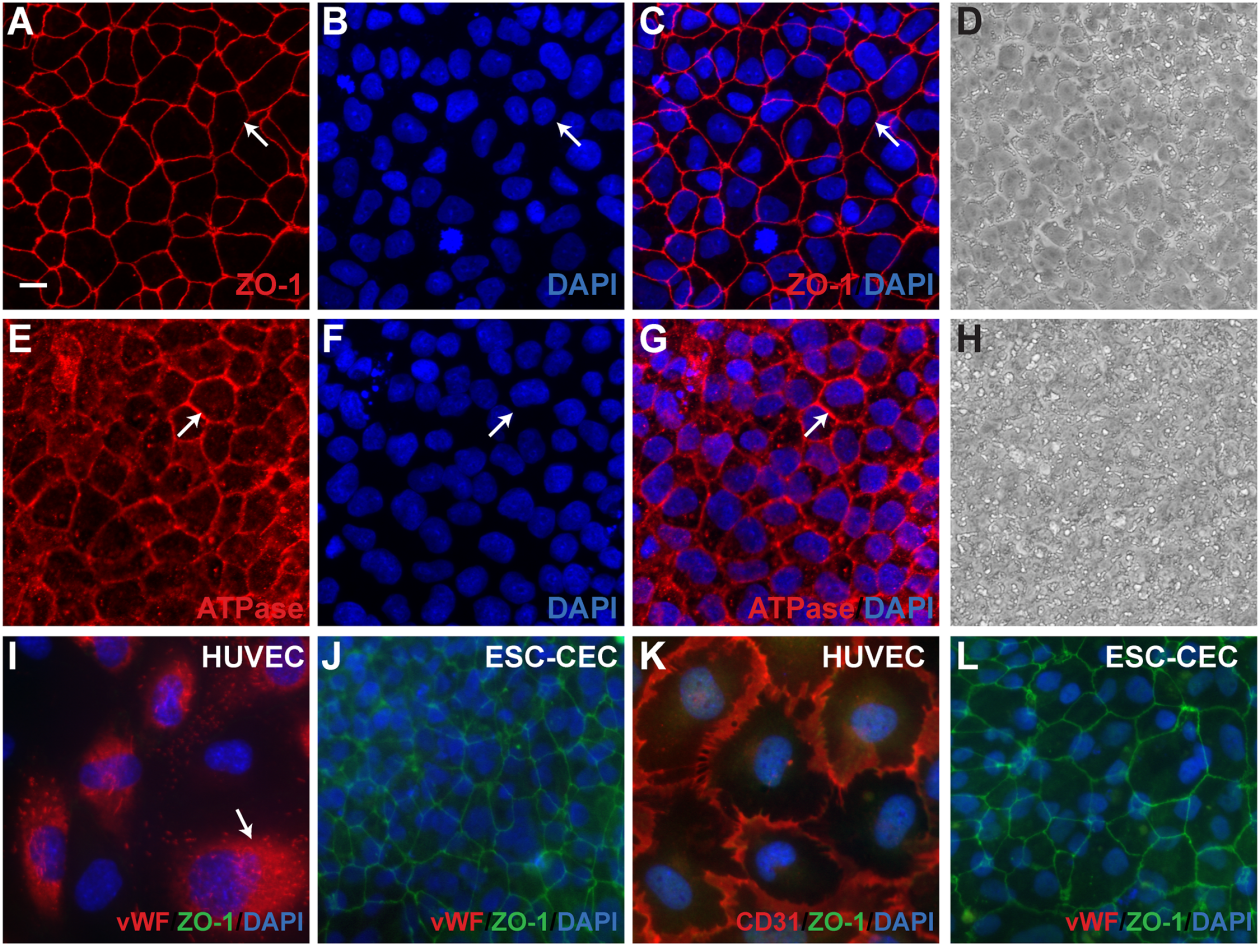
hESC-CEC expressed ZO-1 and Na^+^K^+^ATPaseα1 at the boundaries of cells, but not vascular endothelial markers vWF and CD31. A. Zona Occuldins-1, ZO-1 (red), an adherens tight junction marker, was expressed at the cell borders in hESC derived CEC indicating that the cells were tightly adhered and were hexagonal/polygonal in shape. B. Nuclear marker DAPI (blue) was on a different plane than ZO-1. C. Merge of ZO-1 and DAPI. D. Representative phase contrast picture from same experiment. E. Na^+^K^+^ATPase**α**1 (red) was localized to the cell borders in hESC-CEC indicating that cells had properly localized a component of the endothelial pump function. E. Nuclear marker DAPI (blue) was on a different plane than Na^+^K^+^ATPase**α**1. F. Merge of Na^+^K^+^ATPase**α**1 and DAPI. G. Human umbilical cord vein endothelial cells (HUVEC) expressed von Willebond factor (vWF, red) a marker of vascular endothelial cells, but not ZO-1 (green). H. hESC-CEC expressed ZO-1 (green), but not vWF (red). I. HUVEC expressed Platelet endothelial cell adhesion 1 (PECAM1 or CD31) but not ZO-1 (green). J. hESC-CEC expressed ZO-1 (green), but not CD31 (red). DAPI (blue) stained nucleus. Scale bars = 10 **μ**m.

To further characterize the putative hESC-CEC colonies, these cells were grown on glass coverslips and immunostained for Zona Occludens 1 (Z0-1) a tight junction marker, and Na^+^K^+^ATPase**α**1 (ATP1A1) pump, a major component of the corneal endothelium pump function. hESC-CECs were immunopositive for ZO-1 at the cell boundaries, suggesting that the cells are tightly adherent (Fig 4A–4C). A representative phase contrast image from the same shows the hexagonal like of the hESC-CEC (Fig 4D). Similarly, Na^+^/K^+^ATPase**α**1 was localized to the cell boundaries of the hESC-CECs (Fig 4E–4G), where Na^+^/K^+^ATPase**α**1 must be localized for the CECs to function as a pump. A representative phase contrast image from the same shows the hexagonal like of the hESC-CEC (Fig 4H). From the expression of ZO-1, hESC-CECs appeared tightly packed. To determine the density of the cells, ZO-1 positive cells were manually counted in random fields. The density of the hESC-CECs was 7605 ± 379 cells/ mm^2^ (mean ± SEM), which was higher than the CEC density of human infants (2,987 to 5,624 cells/mm^2^) [26] and well in excess of the low density associated with dysfunction (lower than about 500 to 1000 cells/mm^2^) [27]. These results indicated that sheets of hESC-CEC could be produced at a density well above the level associated with a functional corneal endothelium.

Recently several papers have been published announcing novel cell type specific or groups of markers of corneal endothelial cells including CYYR1^+^/ SLC4A11^+^/COL8A2^+^ [28], Glypican4 and CD200 [29], and Claudin10b^+^/Claudin14^-^ [30]. Importantly, when generating differentiated cell progeny from pluripotent stem cells, the expression of putative markers should also be tested in other cell types including hESCs, iPSCs, and vascular endothelium. Although corneal endothelial cells are derived from neural crest and are neuroepithelial in origin, vascular endothelium shares many of the same markers of corneal endothelium. Typically, this does not interfere with the interpretation of experiments using primary HCECs from adult cornea since the adult cornea is avascular under normal conditions. However, since the hESC-CECs were derived from stem cells, it was necessary to set a robust identification criteria and demonstrate that the hESC-CECs are not vascular endothelial cells, which express many of the same cell adhesion proteins including cadherins and integrins [31,32] that might be useful to distinguish between the CECs and corneal stroma cells. HCEC also express a number of cadherins, including E-Cadherin, N-cadherin, and VE-cadherin [33] which are also expressed by the stroma or epithelium [34–40]. Interestingly, two vascular endothelial markers were absent from corneal endothelial cells under normal conditions, von Willebrand factor (vWF) and platelet endothelial cell adhesion molecule (PECAM/CD31) [31]. We were not able to detect expression of vWF and CD31 in hESC-CECs (Fig 4J and 4L), but these markers were expressed in cultured human umbilical vein endothelial cells (HUVEC) (Fig 4I and 4J). vWF protein had perinuclear localization while CD31 was localized at the cell boundaries of HUVEC cells. HUVEC cells did not express detectable levels of ZO-1 (Fig 4K and 4L), while the hESC-CEC expressed ZO-1 at the cell boundaries (Fig 4J and 4L). Thus, the localization of ZO-1 and Na^+^K^+^ATPase**α**1 to the cell boundaries and the absence of the vascular endothelial cell markers vWF and CD31 in hESC-CECs further support the hypothesis that CECs can be generated from hESCs.

### Descemet’s Membrane Components Are Produced by hESC-CECs

**Fig 5:**
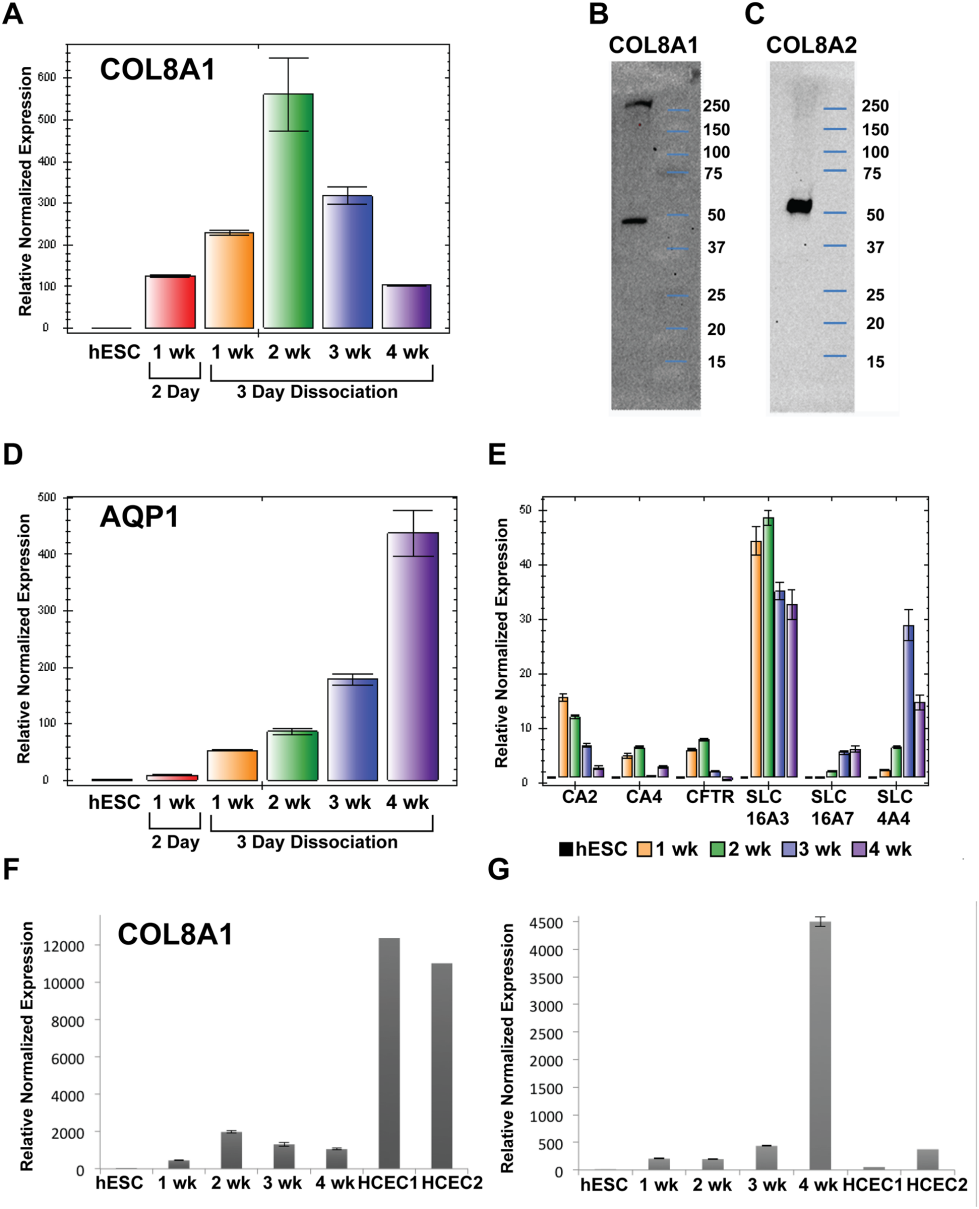
Genes expressed by corneal endothelial cells were present in hESC-CEC. A. COL8A1, a major component of the Descemet’s membrane was expressed is upregulated after 1 week of induction compared to hESC by QPCR utilizing the **ΔΔ**Ct method of analysis and the endogenous control PGK1. mRNA levels peaked around the second week which may have indicated that the cornea endothelial cells no longer need to produce as much COL8A1 to form Descemet’s membrane in vitro. B, C. hESC-CEC secreted COL8A1 and COL8A2 in vitro. The subcellular extracellular matrix secreted by hESC-CEC was analyzed by standard Western blot analysis for the presence of COL8A1 and COL8A2. D. Aquaporin 1 (AQP1) is a water pump that functions as part of the endothelial pump that keeps the cornea dehydrates. The increase in AQP1 expression may indicate increased ability of hESC-CEC to function as a pump. E. Many components of the corneal endothelial pump function were enriched in hESC-CEC. Carbonic Anhydrase 2 (CA2), Carbonic anhydrase 4 (CA4), Cystic Fibrosis Transmembrane conductance receptor (CFTR), Solute Carrier Family 16, member 3(SLC16A3)/Monocarboxylic acid transporter 4 (MCT4), Solute Carrier Family 16, member 7 (SLC16A7)/ Monocarboxylic acid transporter 2 (MCT2), and Solute carrier family 4, sodium bicarbonate cotransporter, member 4 (SLC4A4)/Sodium Bicarbonate cotransporter 1 (MBC1) were enriched in hESC-CECs at 1–4 weeks after induction with the exception of CFTR at 4 weeks, where it was expressed at levels similar to undifferentiated hESCs. F. hESC-CEC weeks 1–4 express about 5 fold less COL8A1 than 2 separate samples of human cultured CEC (HCEC). Normalized to hESC and the endogenous controls of 18S and GAPDH. G. hESC-CEC weeks 1–3 express similar levels of AQP1 as HCEC with the exception of 4 weeks. Normalized to hESC and the endogenous controls of 18S and GAPDH. Wk = week.

Using both day2 and day3-switch culture methods, mRNA was quantified by QPCR to determine the expression of many corneal endothelial genes simultaneously. Expression of COL8A1 was increased 20- to 100-fold over undifferentiated hESCs, with peak expression around 2 weeks in culture (Fig 5A). Interestingly, COL8A1 levels began to decrease after 2 weeks, raising the hypothesis that Col8A1 ECM suppressed ongoing production of COL8A1, a model not tested here. We next assessed the expression of the two isoforms of CEC ECM proteins COL8A1 and COL8A2 in the subcellular matrix. hESC-CECs were grown for 2 weeks to produce the putative *in vitro*-generated Descemet’s membrane. To isolate the subcellular matrix, the cells were removed by exposure to ammonium hydroxide (see Methods). Both COL8A1 and COL8A2 were present in the subcellular matrix by Western blot analysis (Fig 5B and 5C). Both antibodies detected bands around 50 kDa as expected; an additional band detected by COL8A1 antibodies larger than 250 kDA was likely unsolubilized homotrimers of COL8A1 [41]. Thus COL8A1 and COL8A2 presence in the subcellular matrix underneath the hESC-CECs indicates that the hESC-CECs were capable of producing a major component of Descemet’s membrane.

### Corneal Endothelial Pump Components Are Expressed by hESC-CECs

The major component of the corneal endothelial pump function is Na^+^K^+^ATPase**α**1 (ATPA1A). This protein was expressed and localized to the expected location in the hESC-CEC, indicating potential for proper function (Fig 4E). Both Na^+^K^+^ATPase**α**1 and Na^+^K^+^ATPase**α**3 mRNA were highly expressed by hESC-CECs, however, naïve hESCs also expressed Na^+^/K^+^ATPase**α**1 and Na^+^/K^+^ATPase**α**3, which made these two not useful as differentiation markers (data not shown). Similarly, other corneal endothelial pump function genes SLC16A1, SLC9A1, SLC4A2, ADCY10, and CA12 [42–45] were expressed by hESC-CECs, but were not upregulated compared to hESCs (data not shown). On the other hand, aquaporin 1, another vital pump component [46], was expressed by hESC-CECs in increasing levels with time, indicating a maturation of the hESC-CEC (Fig 5D). Other pump proteins detected with varying increases in expression over the 4 week culture period included: carbonic anhydrase 2 (CA2), carbonic anhydrase 4 (CA4), cystic fibrosis transmembrane conductance receptor (CFTR), solute carrier family 16, member 3 (SLC16A3)/monocarboxylic acid transporter 4 (MCT4), solute carrier family 16, member 7 (SLC16A7)/monocarboxylic acid transporter 2 (MCT2), and solute carrier family 4, sodium bicarbonate cotransporter, member 4 (SLC4A4)/sodium bicarbonate cotransporter 1 (MBC1) [42,43,47] (Fig 5E). hESC-CEC did not express the corneal epithelial marker, Keratin 3, nor the stromal markers, Keratocan, and Lumican (data not shown). In addition, mRNA levels of COL8A1 and AQP1 were compared between 2 separate cultures of HCEC and hESC-CEC weeks 1–4. COL8A1 is expressed approximately 5 fold more in HCEC compared to hESC-CEC (Fig 5F). AQP1, on the other hand, was expressed at similar levels between HCEC and hESC-CEC, with the exception of 4 weeks hESC-CEC which expressed significantly higher levels.

### Global Gene Analysis Reveals Close Similarity between hESC-CECs and Adult HCECs

Morphology, mRNA expression, and protein expression and localization were similar in hESC-CECs compared to human CECs. We next expanded on these studies by performing a comparative global gene expression analysis between hESC-CEC and primary cultured human adult CECs (HCECs). Cultured HCECs (passage 0) were chosen over freshly dissected endothelium for several reasons. First, cell culturing itself can change the expression of levels of genes compared to freshly dissected, therefore to properly compare gene expression levels between the cultured hESC-CECs and primary HCECs, the primary cells were subjected to cell culture. Second, the culturing of HCECs before passaging allowed for modest expansion of CEC numbers, so fewer corneas were used. Finally, culturing HCECs allowed for visual inspection to confirm the absence of stromal fibroblasts, an important purity criteria that cannot be guaranteed when peeling off endothelium in cadaveric corneas.

**Fig 6:**
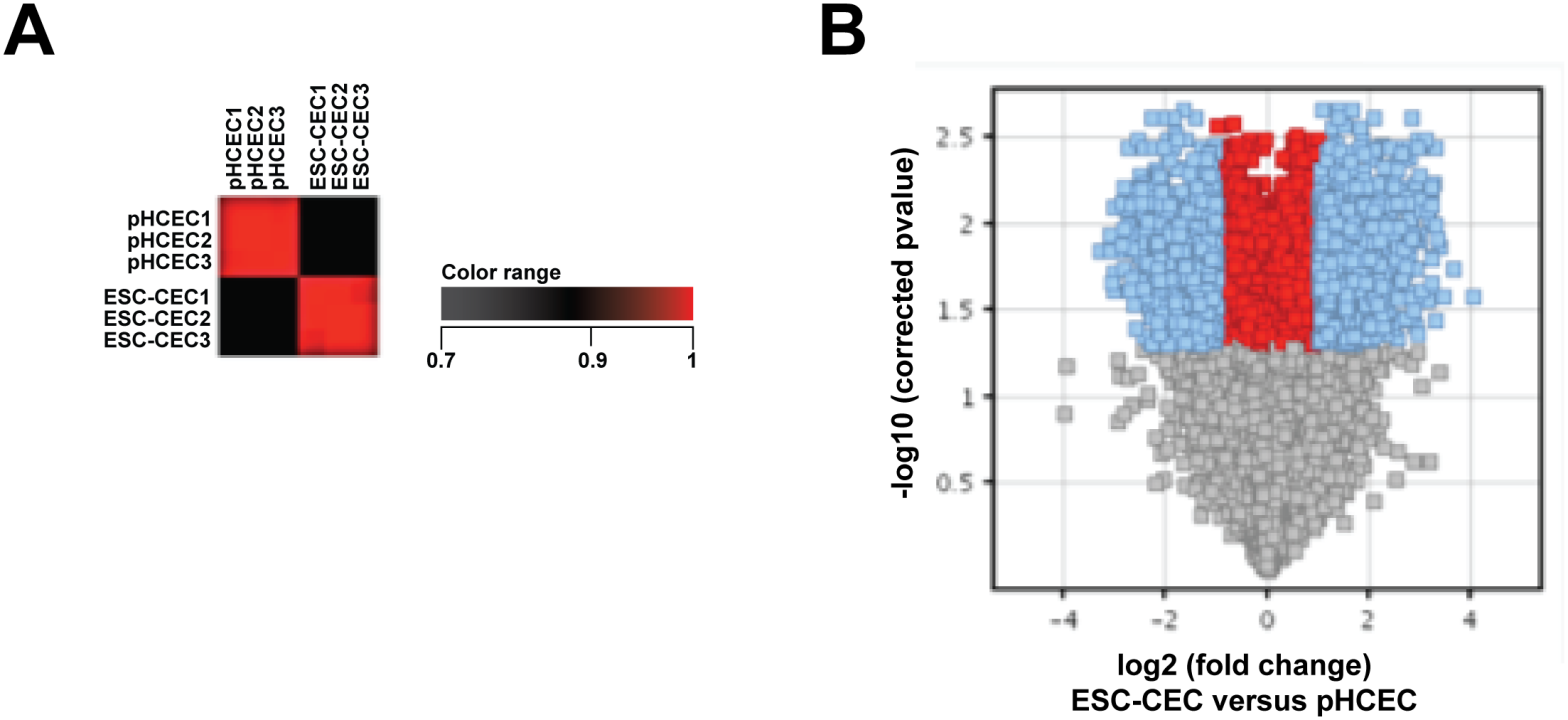
Microarray analysis indicates hESC-CEC were highly similar to primary cultured adult HCECs. A. Correlation analysis. Red color indicated little global variability between samples within group of human corneal endothelial cells (pHCECs) and human embryonic stem cell derived corneal endothelial cells (ESC-CEC). B. Volcano plot indicated most genes expressed by pHCECs and ESC-CEC were within 2 fold.

To perform microarray analysis, total RNA was collected from three independent biological replicates per condition. RNA samples were processed and hybridized to Affymetrix Human Exome ST arrays. Raw data was subsequently normalized and analyzed using Genespring GX12 and Excel software applications. For each condition, the three samples in each condition had a high correlation coefficient (Fig 6A). After normalization, the probes were filtered to only examine those with expression values between the 20^th^ and 99^th^ percentile in at least 2 out 3 biological replicates in at least one cell type. Of the 28869 total probes, 28374 (98%) were used for subsequent analysis. Most probes showed no significant change greater than 2-fold between hESC-CECs and primary HCECs (Fig 6B). Closer statistical comparison using an unpaired T test with p <0.05 (asymptotic p-value computation, multiple testing correction Benjamini-Hochberg) revealed that approximately 1697/28374 (6%) of the probes differed at least 2-fold and 782/28374 (3%) differed at least 3-fold (S1 Table). These findings support the morphological and QPCR data suggesting that hESC-CECs and HCECs were very similar at the level of gene expression. We further explored which pathways were differentially regulated between hESC-CECs and primary HCECs, and interestingly, found that the main over-represented pathways were associated with cell adhesion and cell cycle (S1 Fig).

**Fig 7:**
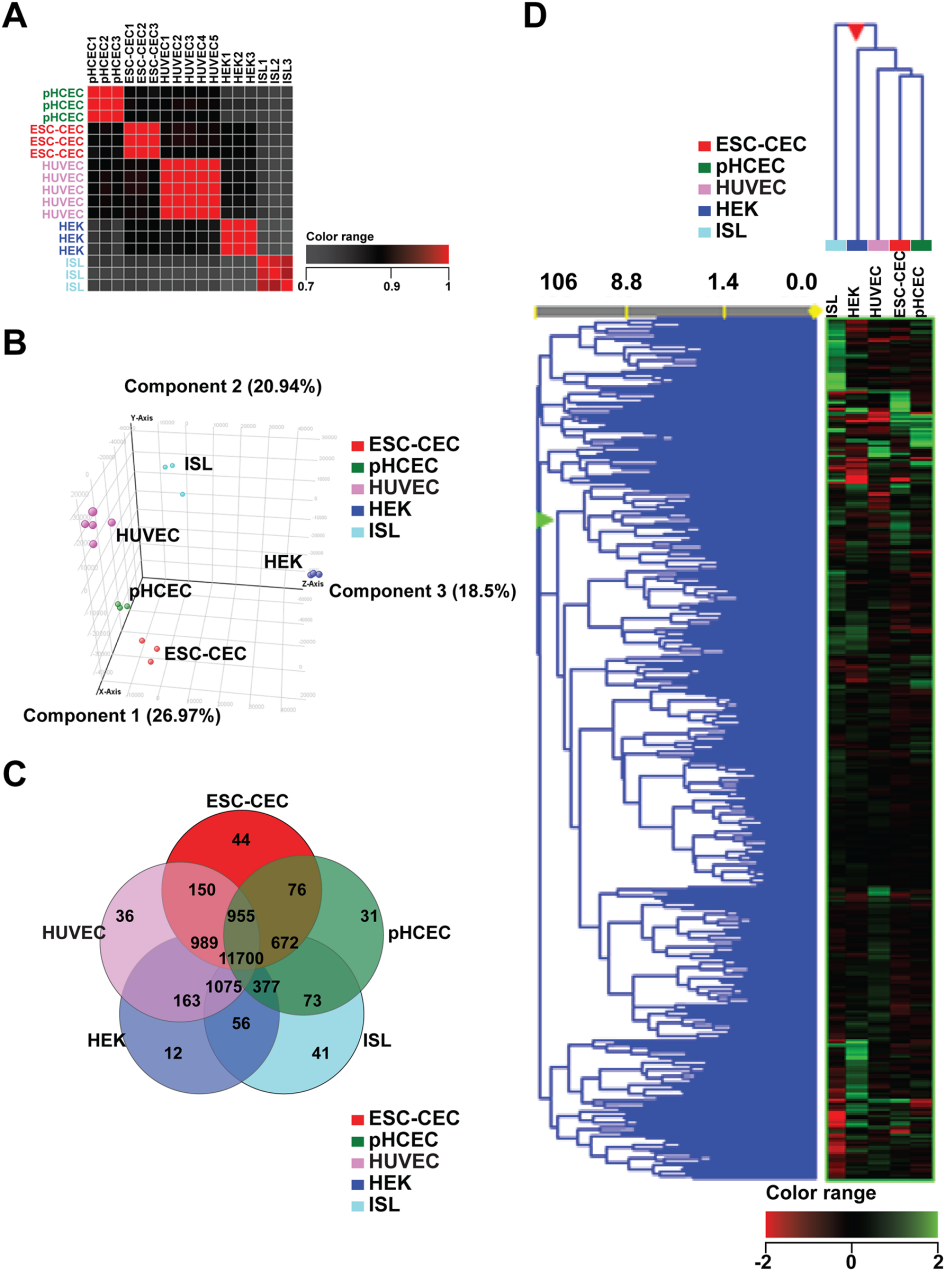
Microarray analysis indicated that hESC-CEC and HCEC have the most similar genetic profile compared to HUVEC, HEK 293, and pancreatic Islet cells. Publicly available datasets for HUVEC, HEK293, and human pancreatic Islet cells were compared to ESC-CEC and pHCEC. A. Correlation analysis indicates that samples within groups have little variation. B. Principal component analysis indicates that ESC-CEC were mostly closely grouped to pHCEC, with HUVEC showing the next closest similarity. HEK 293 (HEK) and pancreatic Islet cells (ISL) were the least similar. C. Venn diagram of total probes. Of note, 76 genes appear to be uniquely expressed by ESC-CECs and HCECs which could be potential novel identifiers for corneal endothelial cells.

To put this comparison into a useful perspective, we extended the analysis to include other known similar and dissimilar cell types, namely human umbilical vascular endothelial cells (HUVEC), known to share many characteristics with CECs [31], and human embryonic kidney cell lines (HEK293) and human primary pancreatic islets cells, which would be expected to have distinct gene expression profiles from hESC-CECs and HCECs. Publicly available raw microarray files (generated using the same platform as the one in this study, Affymetrix’ Human Exome ST arrays) from cultured HUVEC (5 samples), cultured HEK293 (3 samples), and primary (uncultured) human pancreatic islet cells (3 samples) were compared to those of hESC-CECs and HCECs, with normalization across the 5 different cell types’ data sets. The samples in each cell type clustered tightly together and had high correlation coefficients (Fig 7A and 7B). As hypothesized, HUVEC clustered most tightly with HCECs and hESC-CECs. Both HEK 293 and islet cells were dissimilar to the hESC-CECs and HCECs.

Limited by the differences in culture conditions and the complexity of normalizing microarray data across 4 different laboratories, only 11170/28869 (39%) probes fell in the 20-99^th^ percentile of expression for all cell types. Therefore, the transcriptome of each cell type was separately compared to that of hESC-CECs, such that the number of probes analyzed ranged between 18754 (65%) and 19381 (67%) in these pairwise comparisons. Primary HCECs and HUVEC cells were very similar to hESC-CECs: only 2.7% (531/19381) and 3.5% (655/18850) of the probes differed by at least 2-fold (p<0.05, Benjamini-Hochberg corrected). HEK293 and pancreatic islet cells were less similar to hESC-CECs: 5.3% (1016/19141) and 5.4% (1015/18754) of the probes changed at least 2-fold, respectively. To better capture the relationship of these 5 cell types, we separated their gene expression into a Venn diagram, and we found that 44 genes are unique to hESC-CECs and 31 genes are unique to HCECs (Fig 7C). Interestingly, we found 76 unique probes representing 67 genes that may be exclusive to hESC-CECs and HCECs. (S2 Table), providing a pathway for discovering novel markers specific to CECs but not vascular endothelial cells. Hierarchical clustering analysis confirmed that hESC-CECs were most similar to HCECs; HUVEC were the next closest group followed by HEK293s and finally islet cells (Fig 7D).

## Discussion

A potential solution to worldwide limitations in donor corneal tissue and the increasing patient demand for cornea transplant to replace the corneal endothelium [48,49] is to generate corneal endothelium from pluripotent cells. Here we have generated CECs from hESCs with the long-term goal of utilizing these cells for transplantation in cases of corneal endothelial dystrophies. The premise of CEC cultivation and expansion for transplantation into humans was proposed at least as far back as the 1970’s with two reports on the culture of bovine and rabbit CECs [50,51]. However, the corneal endothelium in cows and rabbits can readily regenerate in vivo, and not surprisingly, survive well and proliferate in culture. On the other hand, human and nonhuman primate corneal endothelium does not easily lend itself for in vitro culture or expansion, although it has been investigated over the years. One important barrier may be the lack of high quality corneas for research, as the best human corneas are reserved for clinical transplantation, and non-human primate studies present challenges in access and cost.

When culturing adult human primary corneal endothelial cells, many of the cultures transform into a fibroblastic morphology or fail to proliferate. It has been reported that it is typical of only a third of research grade human corneas generate a viable culture of regular hexagonal cells [52] that can be passaged for a very limited number of times before transforming into fibroblasts. The loss of the endothelial morphological identity is a key indicator for lack of function, as CECs need to form tight junctions to maintain relative dehydration of the cornea. Another challenge is the tendency of the highly proliferative stromal keratocytes to contaminate CEC cultures [6]. Despite these challenges, a number of groups have improved culture conditions [42–55], and have published alternative culture conditions that improve proliferation and limit fibroblastic transformation of primary HCECs [27,56–59]. Here we have taken an alternative approach that allows for a renewable supply of hESC-derived corneal endothelial cells.

The neural crest cells have a tremendous capacity to become a wide variety of cell types from neurons to glia to connective tissue. Therefore it is critical to mimic environmental signals to direct the neural crest to become CECs. Our approach was to first produce neural crest progenitors from hESCs by using dual Smad inhibitors, Noggin and SB431542 [11]. However, we modified their protocol to maximize neural crest progeny before CNS progeny could take over the culture. These young neural crest progenitors were subsequently induced to become corneal endothelium with PDGFB and the Wnt inhibitor/activator Dkk2 in the presence of FGF2. Importantly, these three factors are all present at the right place and time developmentally to induce neural crest to become corneal endothelial cells [60–62]. On the surface, the approximate 7 days of culture to generate corneal endothelial cells seems rapid. However, this timing is consistent with the developing embryonic chicken cornea, where the neural crest cells migrate to the periocular region beginning at embryonic day 3 [63]. The cells wait to enter the area between the lens and presumptive corneal epithelium until embryonic day 4.5. At embryonic day 5, corneal endothelial cells have begun their differentiation. Therefore, we believe it is likely that we may have recapitulated the natural development of CEC from hESC.

A recent report indicated an indirect method of generating corneal endothelial-like cells from hESCs via a cell type that they defined as a periocular mesenchymal precursor (POMP), which has been proposed to have similarities to neural crest [25]. Our approach presented here differs substantially from the POMP method by directing the differentiation of hESC into neural crest and subsequently into CECs by defined factors that are present during development. In this paper, we present the methodology to generate easily scalable quantities of hESC-CECs that rely on a two-step process: directed differentiation of hESCs into neural crest, and then neural crest into hESC-CECs, again with defined factors. The hESC-CECs have been thoroughly characterized by their expression of CEC proteins, secretion of COL8A1 and COL8A2, mRNA expression of known CEC pumps by quantitative PCR (QPCR), and whole genome microarray analysis comparison to primary adult HCECs. We propose that these cells could provide a viable and affordable alternative to the use of cadaveric corneas for corneal endothelial dysfunction.

There are several other reasons why we chose to generate CECs from hESC rather than from human induced pluripotent stem (iPS) cells or adult cadaver-derived HCECs. First, hESC derived cells are currently in testing in the clinic in North America, Europe, and Asia [64–66]. Experimentally, hESC have been traditionally more robust than iPSC with established protocols to expand and maintain karyotypically normal cell lines that are genetically well characterized [67]. By using a feeder-free methodology for cultivating hESCs, hESCs can be split 1:10 every 4–5 days, making it easy to create master cell banks from which to derive hESC-CECs. Newly established iPSC lines are known to have inconsistencies such as incomplete pluripotency and genetically unstability and therefore can lead to variable results in the generation of differentiated cell types. However, iPSC lines have the potential to be used autologously and do not have the ethical concerns associated with the derivation of hESC lines.

Here we have presented the generation of the corneal endothelial cells from human embryonic stem cells. The hESC-CECs are produced by a two step process where first neural crest are generated and then directed to differentiate into corneal endothelial cells using a defined medium with PDGFB, Dkk2, and FGF2. Large fields confluent corneal endothelial cells express Z0-1 and Na^+^K^+^ATPase**α**1 can be generated within 7 days of differentiation from neural crest. Further maturation for a total of 4 weeks shows uniform transformation of the hexagonal cells into the cobblestone morphology associated with human adult corneal endothelial cells. We estimate the purity of the corneal endothelial cells to be between 90–95% based on protein expression and localization of Z0-1, Na^+^/K^+^ATPase**α**1, and COL8A1 as well as mRNA expression of corneal endothelial pump levels versus hESCs. We have been unable to definitively measure the purity of the corneal endothelial cells due to the lack of specific corneal endothelial markers that are not expressed by vascular endothelial cells or hESC. This issue of purity and functionality is also of concern for adult CEC and is beginning to be addressed by the utilization of several CD markers [68] as well as 5 novel markers CLRN1, MRGPRX3, HTR1D, GRIP1 and ZP4, with no documented function in corneal endothelium [69]. Corneal endothelial cells have endothelial properties, but are neural epithelial in origin. Another avenue for future exploration is the potential use of the epithelial markers of Keratin 8 and 23.

There are several potential sources of impurity including undifferentiated hESC, undifferentiated neural crest, or other differentiated cell types. We believe the major source of impurity to be from undifferentiated neural crest as QPCR results indicate a dramatic drop-off of hESC markers Nanog, after 2 days of neural crest differentiation (Fig 1G, [11]). Neural crest makers p75 and Sox10 mRNA are still present after week of differentiation under the original protocol (Fig 1B), however, NGFR/p75 may also be expressed by adult corneal endothelial cells [23]. By modifying the original protocol, we were able to dramatically reduce the amount of undifferentiated cells by dissociating at day 3, which helped convert more cells into corneal endothelial like cells. We have taken two other additional approaches to reducing the number of undifferentiated neural crest, mechanical dissection and maturation. Mechanical dissection is very labor intensive and has a tendency to transform neighboring CECs into fibroblasts as they expand into the newly open space. Interestingly, the undifferentiated neural crest that appear to form in clumps die off with time, leaving sheets of corneal endothelial cells within a few weeks of culture. Other evidence for the 90–95% purity of the hESC-CEC can be found in the microarray analysis of global gene expression is 94% of genes are expressed at similar levels between hESC-CEC and adult CEC. As additional markers for corneal endothelial cells are identified, it may be possible to definitively measure the purity of the hESC-CEC cultures as we continue to improve the culturing techniques. The next step will be to test the hESC-CEC in an animal model for corneal endothelial dystrophy.

Another laboratory has generated corneal endothelial like cells from human embryonic stem cells via cell type that they defined as a periocular mesenchymal precursor (POMP), which has similarities to a neural crest cell [25]. Their approach appeared to generate corneal endothelial like cells, but has several distinct disadvantages. The POMP protocol relied on the spontaneous differentiation of mesenchymal cells derived from an embryoid body [25]. These cells are thought to be similar to neural crest, but the methodology used has not been extensively tested as the Chambers method for neural crest generation [70–72]. This adds unnecessary risk as cell differentiation into neural crest is not well defined by the POMP methodology. Second, the mesenchymal cells are then isolated and exposed to a series of undefined components found in conditioned medium from corneal stromal cells and lens epithelial cells. In the protocol presented here, we utilize defined medium with factors that are known to be present in the cornea during development [60–62]. Third, the authors speculate that intensive work will be required to generate sufficient quantity and purity of these hESC-derived corneal endothelial-like cells for transplantation. Figures show that only patches of corneal endothelial cells can grow. The work presented here deliberately sought out a simple and robust methodology that can be easily adapted and scaled using good manufacturing practices. For these reasons, we believe that our unique differentiation protocol and thorough characterization of the hESC derived corneal endothelium present an important step in the quest to generate an off the shelf corneal endothelium for transplantation.

## Conclusion

Cornea transplantation is one of the most successful organ transplantations with a relatively low risk of rejection. The gold standard of care for corneal endothelial disease is hampered by a lack of donor corneas, leaving many patients with visual defects and blindness. The ability to produce high quality corneal endothelial cells from human embryonic stem cells would improve access to cornea transplantation worldwide. Here we present a simple and robust method for generating scalable amounts of hESC-CEC for transplantation. We have characterized the morphology, localization and expression of key corneal endothelial proteins, and gene expression by QPCR and microarray analysis indicating that the hESC-CEC were highly similar to adult CEC. Although beyond the scope of this paper, future experiments include the transplantation of the hESC-CEC in animal models of corneal edema to test the function of the hESC-CEC. hESC-derived CEC could provide a viable alternative to the use of donor corneas for corneal endothelial cell disease.

## Supporting Information

S1 Fig Over-represented pathways that differ amongst CECs. Pathway analysis of all probes that differed significantly between hESC-CECs and primary HCECs revealed that the main pathways over-represented were related to cell adhesion and cytoskeleton remodeling (p<0.05). The right column represents the number of genes in the dataset that were significantly enriched over the total number of genes in the pathway category as labeled.
(TIF)

S1 Table Differences between HCECs and CECs. List of all probes that differed significantly between hESC-CECs and HCECs by microarray analysis (unpaired T test, p <0.05; asymptotic p-value computation, Benjamini-Hochberg multiple testing correction).
(XLS)

S2 Table List of 67 genes that may be exclusively expressed by HCECs and hESC-CECs, but not HuVEC. 14 of these genes (20%) differed significantly amongst HCECs and hESC-CECs (Fold change > = 2; p<0.05).
(XLSX)
