# Supplementary figures and images for: Efficient Generation of Human Embryonic Stem Cell-Derived Corneal Endothelial Cells by Directed Differentiation
Source: PLoS One. 2015 Dec 21;10(12):e0145266. doi: 10.1371/journal.pone.0145266 (PMC4686926; doi:10.1371/journal.pone.0145266)

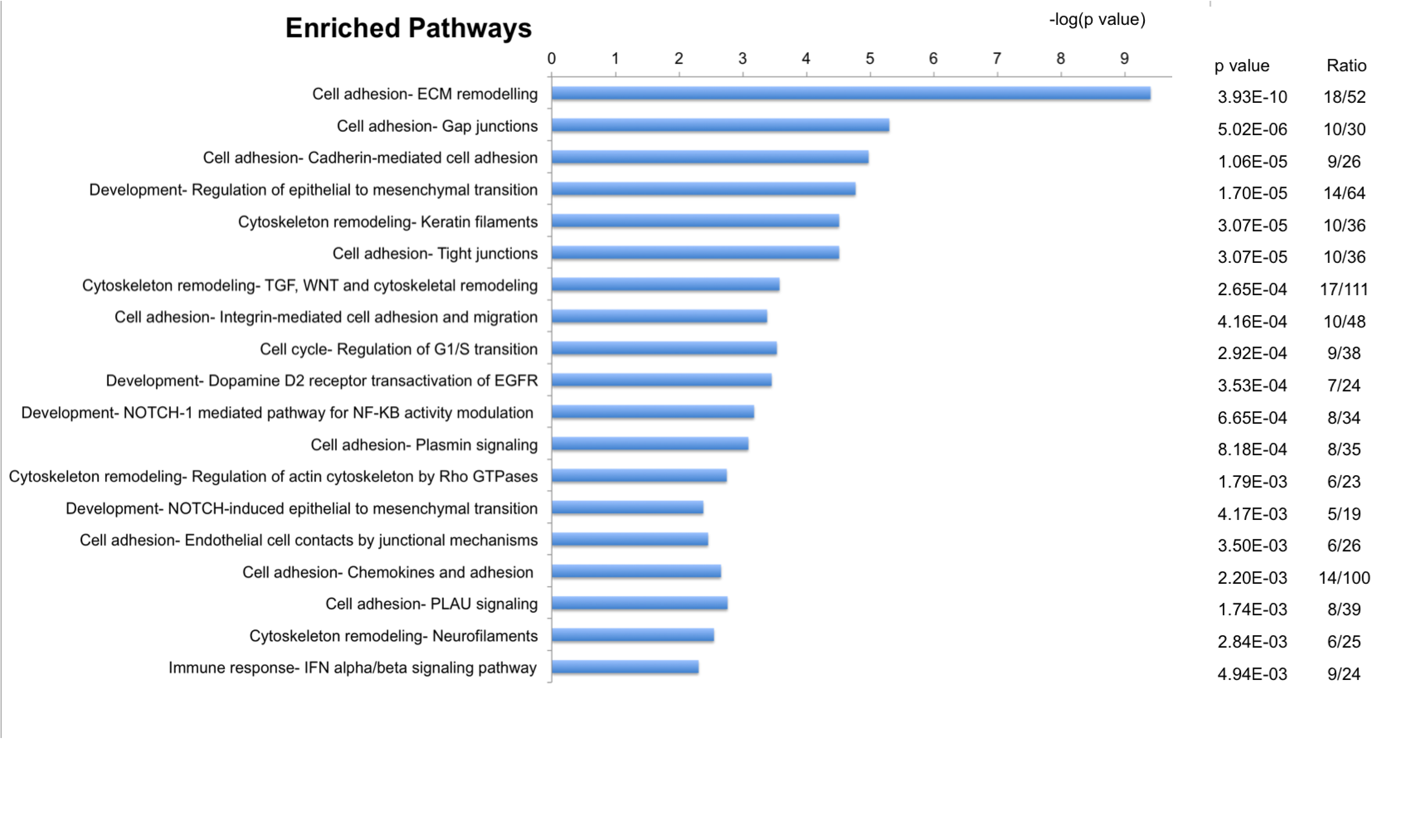

Supplement: S1 Fig — Pathway analysis of all probes that differed significantly between hESC-CECs and primary HCECs revealed that the main pathways over-represented were related to cell adhesion and cytoskeleton remodeling (p<0.05). The right column represents the number of genes in the dataset that were significantly enriched over the total number of genes in the pathway category as labeled. (TIF) [file pone.0145266.s001.tif]
